# Supplementary material for: Valence band engineering of GaAsBi for low noise avalanche photodiodes
Source: Nat Commun. 2021 Aug 6;12:4784. doi: 10.1038/s41467-021-24966-0 (PMC8346614; doi:10.1038/s41467-021-24966-0)
Supplement: Supplementary file 1 — Supplementary Information [file 41467_2021_24966_MOESM1_ESM.pdf]

Supplementary information

Valence Band Engineering of GaAsBi for Low Noise Avalanche Photodiodes

Yuchen Liu[1], Xin Yi[1,2], Nicholas J. Bailey[1], Zhize Zhou[1,3], Thomas B. O. Rockett[1], Leh W. Lim [1], Chee Hing Tan[1], Robert D. Richards[1] and John P.R. David[1,*]

[1]Department of Electronic and Electrical Engineering, University of Sheffield, Sheffield, S1 3JD, UK.

[2]Present address: Institute of Photonics and Quantum Sciences, School of Engineering and Physical Sciences, David Brewster Building, Heriot-Watt University, Edinburgh, EH14 4AS, UK.

[3]Present address: State Key Laboratory of CAD&CG, Zhejiang University, Hangzhou, Zhejiang Province, 310003, P.R. China.

[*]Correspondence should be addressed to J.P.R.D. (email: j.p.david@sheffield.ac.uk)

I. Sample growth

All the devices described in this work were grown using an Omicron molecular beam epitaxy-scanning tunneling microscope system.

The substrate temperatures and cell fluxes were calibrated using reflected high energy electron diffraction measurements of the surface reconstruction[1]. The growth parameter details specific to each device are laid out in Table S1. The common parts of the growth procedure are described here. Each on-axis, GaAs 001 substrate was cleaved to 11.3 × 11.8 mm^2 to fit the substrate mounting mechanism. Upon loading into the growth chamber, the substrates were outgassed at ~ 400 °C for 20 minutes to remove any volatile chemicals from the surface, then raised to 600 °C under an As_2 flux in order to remove the native oxide from the growth surface. A doped GaAs layer was grown at ~ 580 °C under a Ga:As_2 atomic flux ratio of ~1.4. For all devices, Be was used as a p-type dopant and Si was used as an n-type dopant. Following growth of the lower cladding layer, the substrate temperature was dropped to the GaAsBi growth temperature and the As flux was changed from As_2 to As_4 near stoichiometry during a 20-minute growth pause. Prior to growth of GaAsBi, Bi was deposited on the surface in the absence of a Ga flux for ~30 s. This Bi pre-layer was intended to populate the growing surface with Bi in order to reduce the time taken to reach equilibrium when growing GaAsBi. Following GaAsBi growth the substrate temperature was increased to 580 °C and the As species changed back to As_2 during another 20-minute growth pause for the upper cladding region growth. The doping of the final ~ 10 nm of the upper cladding layer was increased by reducing the Ga flux towards the end of the cladding region growth in order to produce a good contact layer.

Supplementary Table S1 Sample growth information.

Diode type	*Layer No.*	*Nominal i-region thickness (nm)*	*Growth rate (μmh⁻¹)*	*GaAsBi growth T (°C)*	*Bi flux reading from ion gauge (nA)*
$p-i-n$	**P1**	200	0.63	378	0.78
	**P2**	400	0.61	378	0.84
	**P3**	800	0.61	378	0.84
	**P4**	1600	0.61	378	0.84
	**P5**	400	0.56	410	1.7
	**P6**	400	0.56	365	1.6
$n-i-p$	**N1**	200	0.42	357	0.72
	**N2**	400	0.42	357	0.72
	**N3**	800	0.41	357	0.35
	**N4**	400	0.29	395	0.24
	**N5**	400	0.30	395	0.27
	**N6**	400	0.30	395	0.35
	**N7**	200	0.39	360	1.0

II. Device fabrication

The devices were fabricated by standard photolithography and wet chemical etching, using a 1:1:1 mixture of hydrobromic acid, acetic acid and potassium dichromate to etch circular mesa diodes with diameters of 50, 100, 200 and 400 μm. In/Ge eutectic and Au was used for the n+ metal contact and Au-Zn-Au was used to form the p+ contact. A schematic diagram of the device structure and plan view image of fabricated devices is shown in Fig. S1.

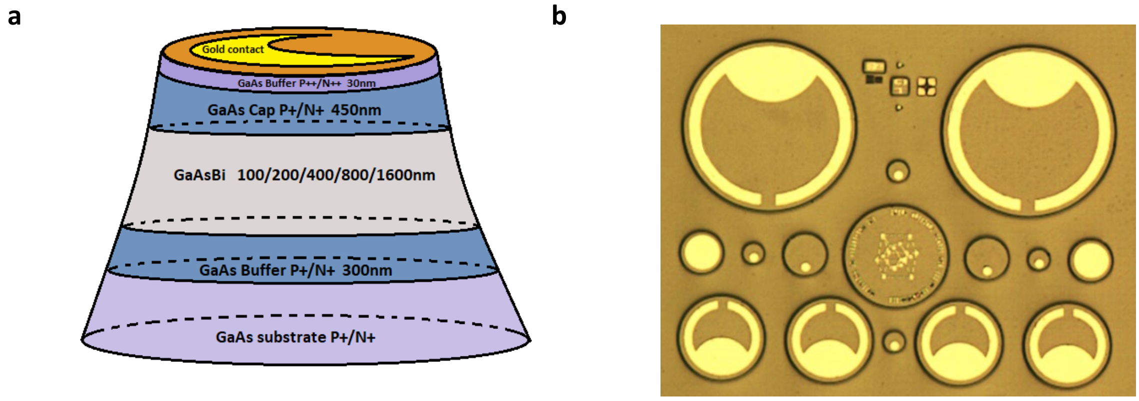

Fig. S1 a, Schematic diagram of device structure. **b**, Plan view image of devices after metallization and etching.

III. Capacitance-voltage measurement

a **b**

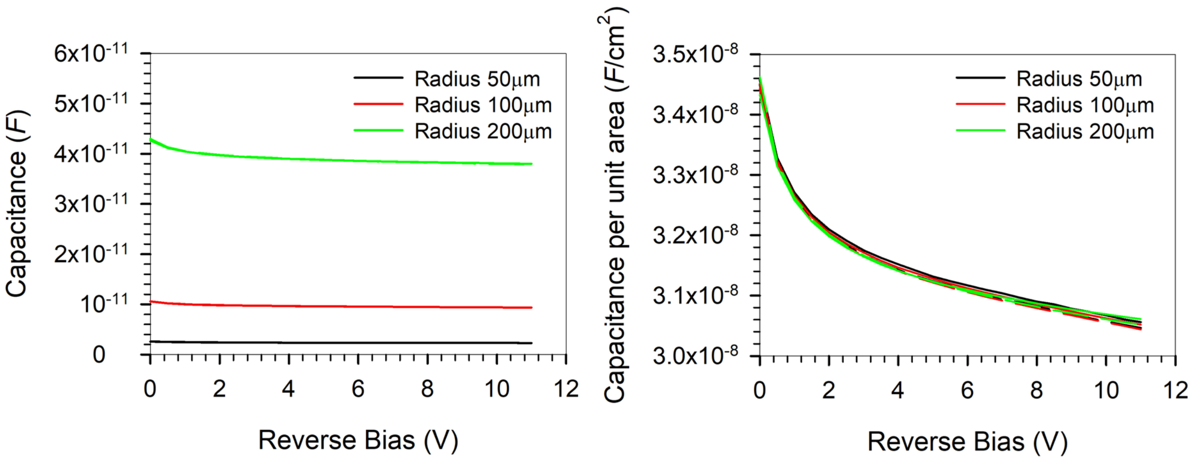

Fig. S2 a, Capacitance-voltage results for different radii devices from layer N2. **b,** Capacitance per unit area as a function of the reverse bias for N2.

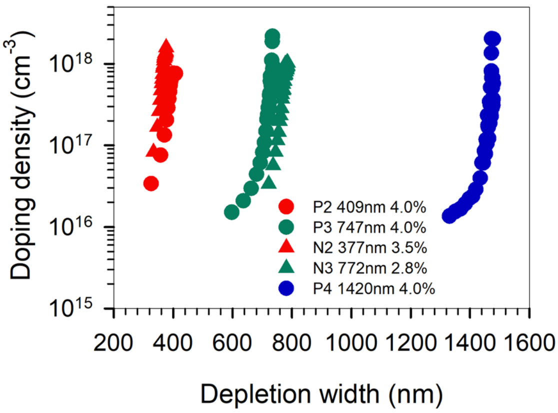

Fig. S3 Doping densities of P2, N2, P3, P4 and N3.

Capacitance-voltage (CV) measurements were undertaken using an HP4275A LCR meter at 1 MHz. Figure S2 **a** shows representative CV results for different radii devices for layer N2 and Fig. S2 **b** shows that these scale with the device area. A static dielectric constant (ε_r) of 12.95 (similar to that of GaAs) was used to determine the depletion thickness of the i-region and the doping profile. An assumption was made that the n^+ silicon doping in the n-GaAs cladding region is higher than the p+ Be doping in the p-GaAs cladding region. Fig. S3 shows the doping profile obtained for devices from P2, P3, P4, N2 and N3. The effect of Debye blurring makes the doping at the interface appear less abrupt than it actually is. The intrinsic thickness of each sample was estimated as the distance when the doping density was ~ 1×10^{18} cm^{-3}. The fact that P4 shows a depletion width of ~1300 nm with just the built-in voltage of ~ 1.2 V suggests that the doping level in the i-regions must be < 10^{15} cm^{-3}.

IV. X-ray diffraction (XRD) spectrum simulation

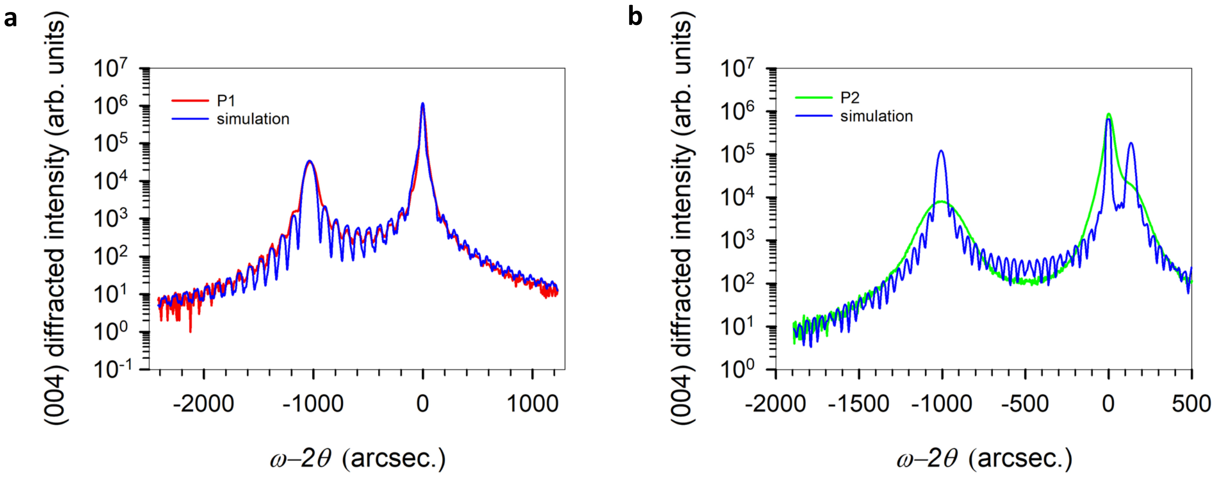

Fig. S4 a(b), 004 XRD ω-2ϑ pattern of P1 (P2). The solid blue lines are simulations using the RADs mercury software.

For XRD, 004 ω-2ϑ scans were performed on each device using the Cu Kα_1 line with a Bruker D8 Discover. The spectra were fit using Bede RADS Mercury software. In each case, the Bi content in the GaAsBi layer was assumed to be uniform and the GaBi lattice constant was assumed to be 6.324 Å[2]. Several of the devices (P1, N4, N5) show clear interference fringes and yield simple, accurate fits (see Fig. S4 **a**); however, the devices with a thicker GaAsBi layer or a higher Bi content show evidence of strain relaxation, which manifests as a loss of the interference fringes and the appearance of a tensile GaAs peak due to the growth of the GaAs cladding on a GaAsBi virtual substrate (see Fig. S4 **b**). In these instances it is impossible to uniquely define the Bi content by fitting the spectrum, as the GaAsBi composition and relaxation, as well as the GaAs cap relaxation, determine the locations of the two peaks. On these occasions the XRD data was reconciled with bandgap data to determine the Bi content and relaxation of the GaAsBi layers, as described in the Bi content determination section. For verification of the results, selected devices were sent to Warwick Scientific Services, who produced reciprocal space maps of the 004 and 224 reflections to uniquely define the Bi content and relaxation. These analyses produced results that agreed with our results to within approximately ± 0.1 % Bi.

V. Photocurrent spectral response

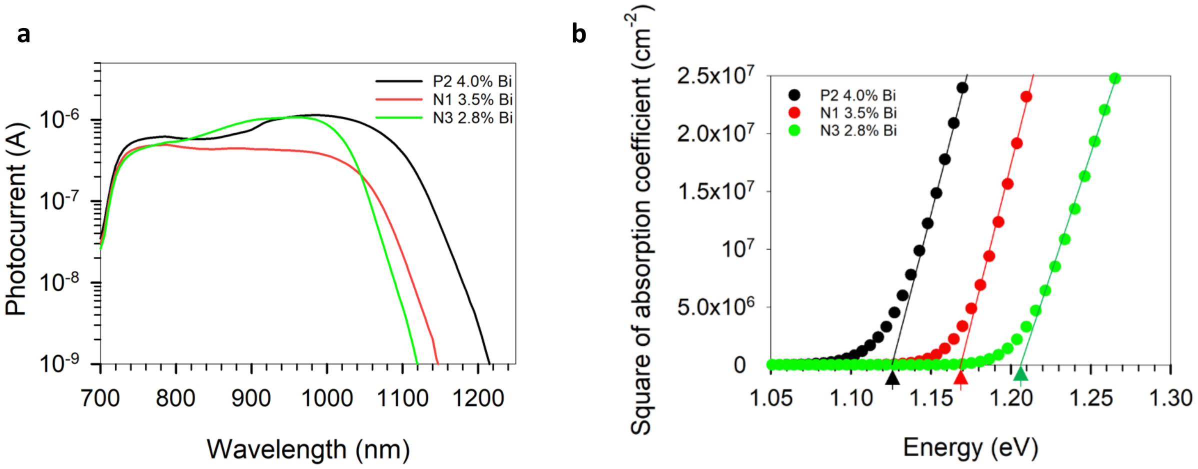

Fig. S5 a, Photocurrent spectra from P2, N1 and N3. **b,** Square of the absorption coefficient of P2, N1 and N3. The bandgap energy of each layer (indicated by an arrow) was estimated from the linear region's extrapolated x-axis intercept.

Room-temperature photocurrent measurements were undertaken on the diodes using a tungsten-lamp, a grating monochromator and a lock-in-amplifier. Fig. S5 **a** shows an example of the measured photocurrent at 0 V obtained on 200 µm radii devices from P2, N1 and N3. As the Bi content increases, the cut-off wavelength redshifts as expected. By normalising the photocurrent, using calibrated Silicon and InGaAs photodiodes, a device's quantum efficiency and absorption coefficient can be determined. From the absorption coefficient, the direct bandgap (E_g) can be obtained by using the following expression[3]:

$$a(h\omega) \propto (h\omega - E_g)^{1/2} \qquad (1)$$

Where h is Planck's constant and ω is the frequency of incident photons. The device's bandgap can now be estimated by plotting the square of the absorption coefficient against incident photon energy as shown in Fig. S5 **b** for these three layers.

VI. Determination of Bi content

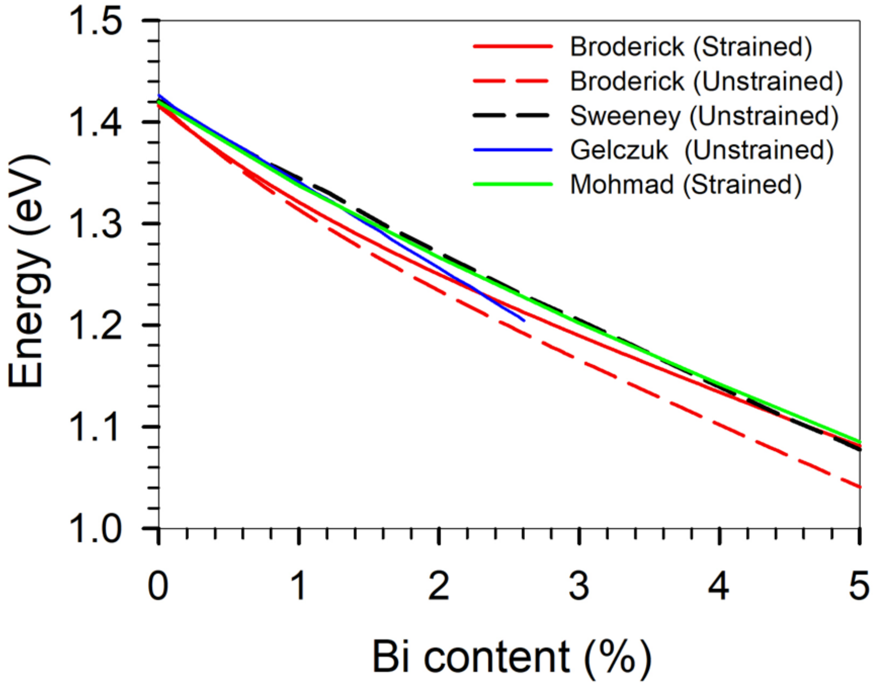

Fig. S6 Energy of the fundamental bandgap as a function of Bi composition for unstrained and strained GaAsBi, reported by different groups.

From the literature, different research groups have reported the relationship between bandgap energy and Bi content as shown in Fig. S6. Broderick et al.[4] undertook theoretical calculations for strained and unstrained GaAsBi using a 12-band k•P model as shown by the solid and dashed red lines respectively. Usman et al.[5] undertook a full tight-binding calculation for strained GaAsBi and found that the bandgap energy was almost identical to that of Broderick et al. for the range of Bi% considered here (not shown for clarity). Fig S6 also shows the predicted bandgap versus Bi content using the valence band anti-crossing (VBAC) model combined with the virtual crystal approximation (VCA) as determined by Sweeney et al.[6], Gelczuk et al.[7] and Mohmad et al[8]. There are some differences in these results, possibly due to the different values of coupling parameters that were used.

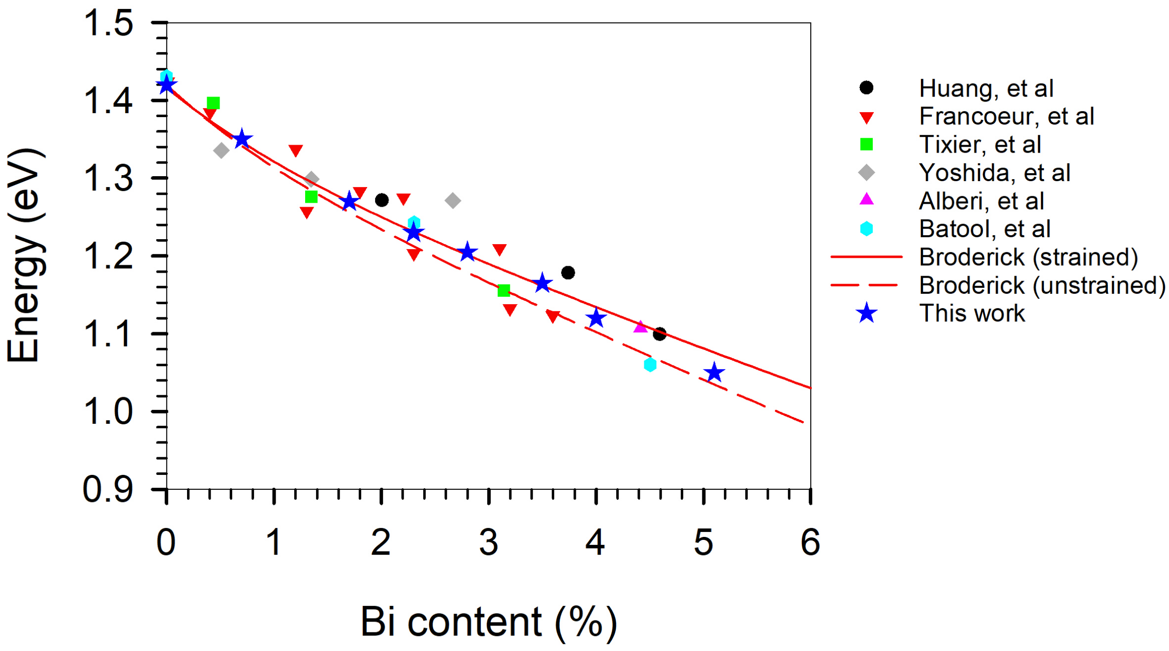

Fig. S7 Predicted bandgap (E_g) plotted as a function of Bi content for strained and unstrained GaAsBi. Experimental results from this work and six other reports are also plotted for comparison.

Fig. S7 shows the relationship between bandgap energy and Bi content for experimental data from the literature and this work. Only the theoretical model of strained and unstrained GaAsBi proposed by Broderick et al[4] is plotted in the graph for clarity.

The bandgap energies stated by Huang et al.[9] and Tixier et al.[10] were obtained by photoluminescence, while the bandgap energies stated by Francoeur et al.[11], Yoshida et al.[12], Alberi et al.[13] and Batool et al.[14] were obtained either by modulated electroreflectance or photo-reflectance spectroscopy. The Bi content was determined either from XRD[10,14] or Rutherford Back Scattering[9,11,12,13]. In this work, photocurrent spectral response measurements were used to determine the bandgap energy (see the Photocurrent spectral response section) and the Bi content was obtained from 004 XRD measurements (see the X-ray diffraction (XRD) spectrum simulation section). Excellent agreement was achieved with the theoretical strained line of Broderick et al[4] for the thinner samples with Bi content less than 3.5 % while for the thicker or higher Bi content samples, where appreciable strain relaxation is known to occur, the data tends to agree more with the unstrained line. This enables us to have a high degree of confidence in the values of Bi content attributed to the samples in Table 1 of the main paper.

VII. Multiplication and excess noise

Multiplication and excess noise measurements were performed on the GaAsBi diodes using the noise measurement setup, as shown in Fig. S8. The center frequency of the noise system was 10 MHz with a bandwidth of 4.2 MHz. Two lock-in amplifiers were used, which allowed us to measure the photocurrent and excess noise of the devices simultaneously and to distinguish the real signal from any leakage or dark current. The excess noise factor, *F*, was calculated using the equation

$$F = \frac{N_c}{k_s I_{ph} M^2} \quad (2)$$

where I_{ph} is the unmultiplied primary photocurrent of the device under test, M is the multiplication factor, and N_c and k_s are the corrected noise power and the output noise power per unit input photocurrent of the noise measurement system. The N_c and k_s parameters were calculated from a commercial silicon photodiode (BPX65). Further details of the measurement system used are given by Lau et al[15]. In order to ensure the reproducibility of results, several devices were measured on each sample.

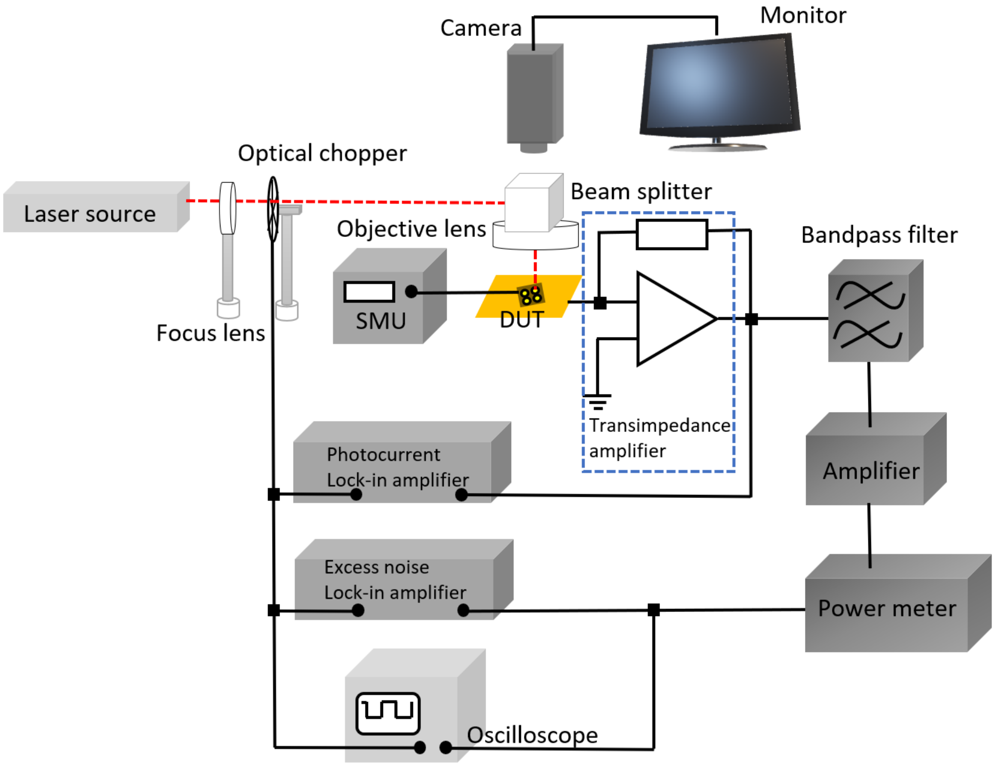

Fig. S8 The measurement setup used to obtain the multiplication and excess noise data. DUT denotes the device under test and SMU denotes the source measure unit.

VIII. Extraction of alpha and beta for different Bi content

Extraction of the ionization coefficients requires that the bias dependence of the photocurrent is obtained. This was done using a lock-in amplifier and phase sensitive detection (to remove the contributions of the dark currents). Typical examples of the un-normalised photocurrents are shown below in Fig. S9 for the p-i-n and n-i-p structures. Any small increase in the photocurrent prior to the onset of avalanche multiplication was corrected for as described by Woods et al [16] to give us M_e and M_h.

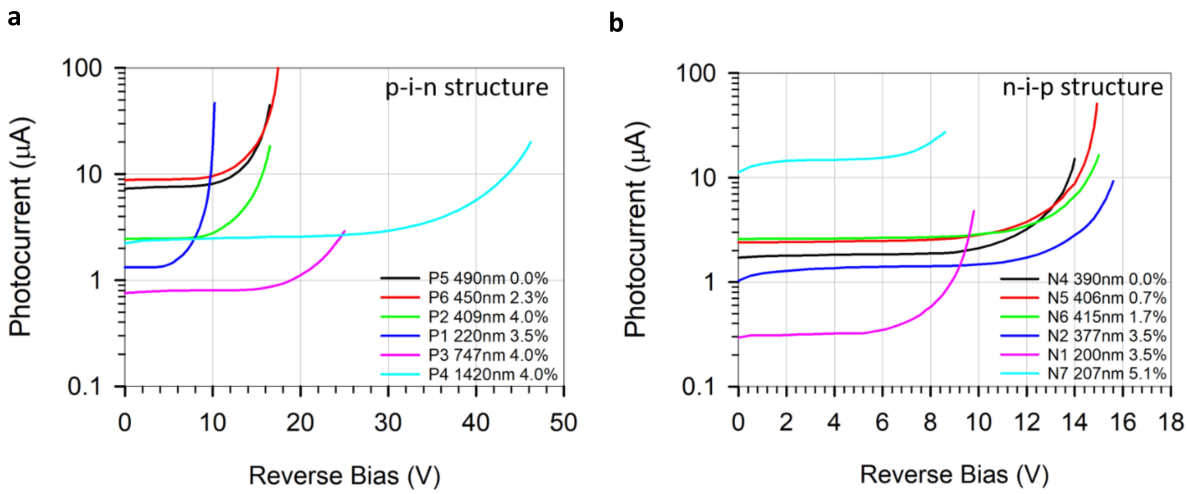

Fig. S9 a, Measured photocurrent from p-i-n structure. **b,** Measured photocurrent from n-i-p structure.

Wavelength-dependent multiplication measurements show that $\alpha > \beta$ in GaAsBi, and so using M_e alone can give an initial accurate indication of α at low electric fields[17]. Using p-i-n diodes with similar Bi contents and different intrinsic region thicknesses enabled the behaviour of α to be estimated over a wide range of electric fields for the 4.0 % Bi composition. This value of α is found to be very close to that of GaAs and so we assume that for arbitrary values of Bi from 0 to 5.1 %, we can safely interpolate or extrapolate values of α as an initial guess. We next estimate the β values from the M_h values of the 0.7 %, 1.7 %, 2.8 %, 3.5 % and 5.1 % n-i-ps. Next, we interpolate (or extrapolate) to estimate the α and β of compositions that we do not have, so we have an initial starting point for α and β of all the different GaAsBi compositions investigated in this work. If we have M_e or M_h from a p-i-n or n-i-p respectively, the larger values of multiplication will involve both α and β as shown by equations 2b and 2c in the main paper. An iterative technique was then used to adjust the values of α and β for each Bi composition until good fits to M_e-1 and M_h-1 were obtained for all of the p-i-ns and n-i-ps (described in Table 1 of the main text; reproduced in Table S2 here), as shown below in Fig. S10. When we have good agreement between the experimental results and the simulated multiplication over a wide dynamic range, we can be confident of the accuracy of the ionization coefficients used.

Supplementary Table S2 Experimental layer details. GaAsBi p-i-n and n-i-p layer details.

Diode type	Layer number	Nominal intrinsic region thickness (nm)	Actual intrinsic region thickness (nm) ± 10 nm	Bi content (%) ± 0.1 %
p – i – n	P1	200	220	3.5
	P2	400	409	4.0
	P3	800	747	4.0
	P4	1600	1420	4.0
	P5	400	490	0
	P6	400	450	2.3
n – i – p	N1	200	200	3.5
	N2	400	377	3.5
	N3	800	772	2.8
	N4	400	390	0
	N5	400	406	0.7
	N6	400	415	1.7
	N7	200	207	5.1

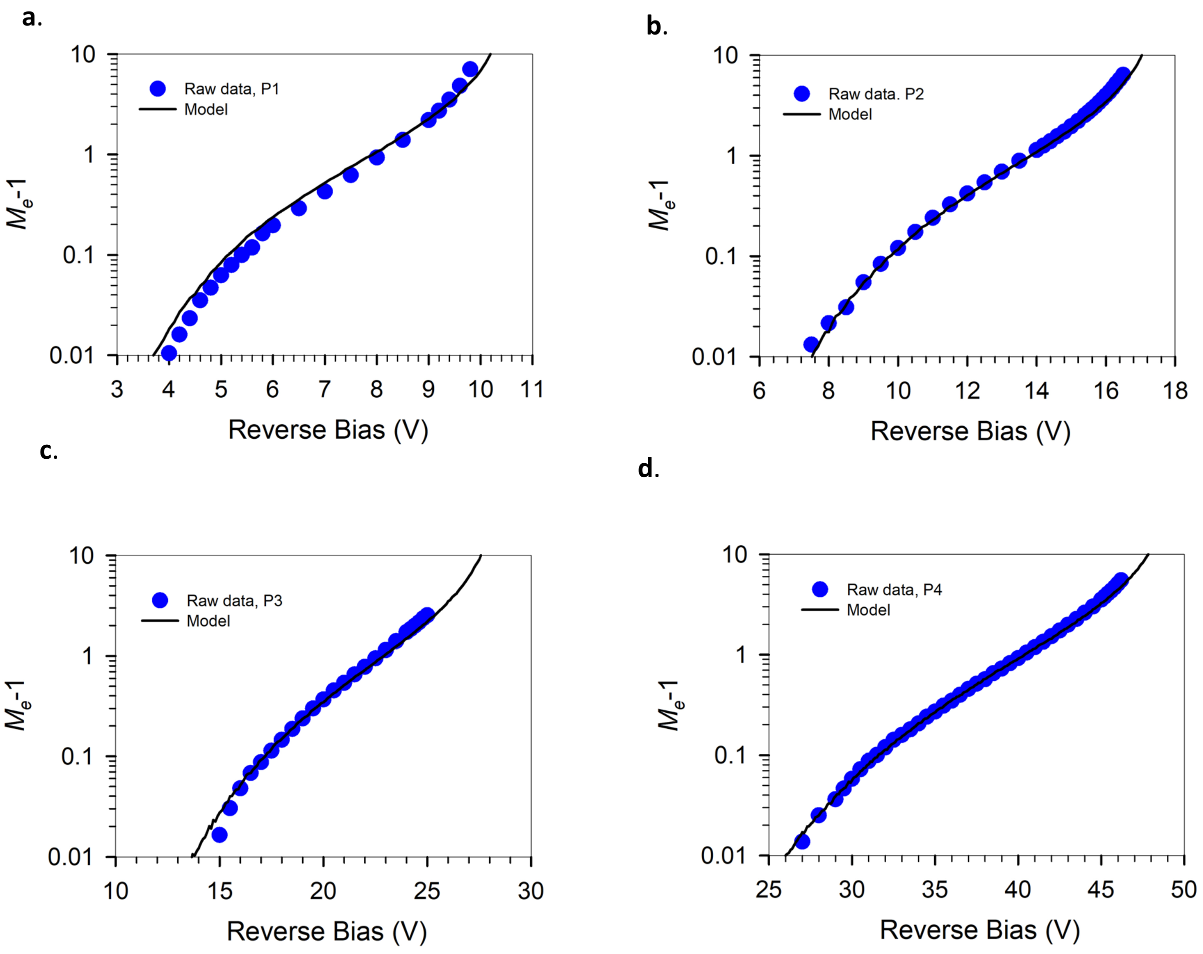

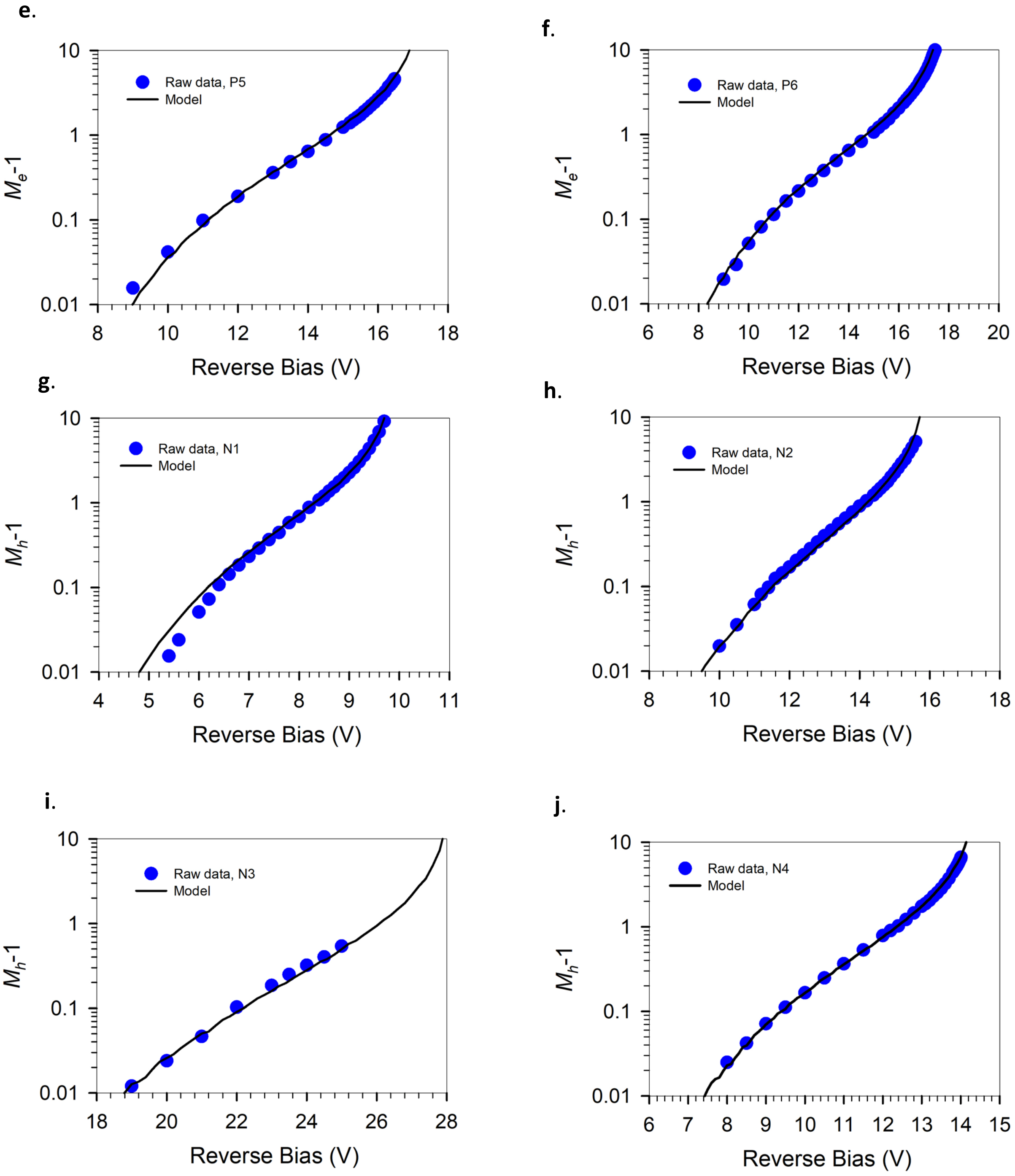
e.
Raw data, P5
Model
M_e-1
Reverse Bias (V)
f.
Raw data, P6
Model
M_e-1
Reverse Bias (V)
g.
Raw data, N1
Model
M_h-1
Reverse Bias (V)
h.
Raw data, N2
Model
M_h-1
Reverse Bias (V)
i.
Raw data, N3
Model
M_h-1
Reverse Bias (V)
j.
Raw data, N4
Model
M_h-1
Reverse Bias (V)

k.

l.

m.

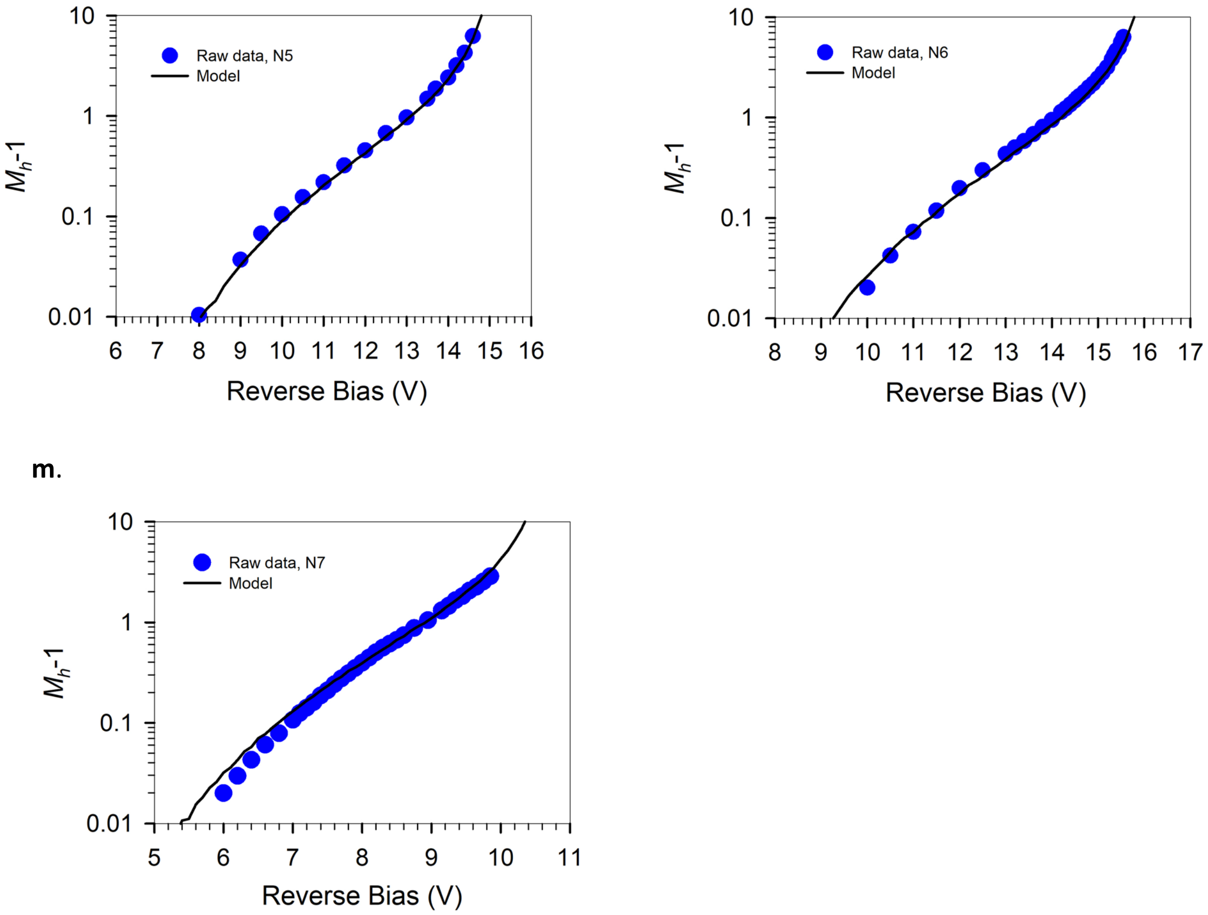

Fig. S10 a-m, Experimentally obtained $M_e(M_h)$-1 (symbols) and modelled (line) for all samples shown in the table S2.

Fig. S11 shows the ionization coefficients obtained by this fitting at an inverse electric field of 3×10^{-6} cm/V. The data show that β (closed triangles) decreases rapidly with increasing Bi content when compared to α (closed circles).

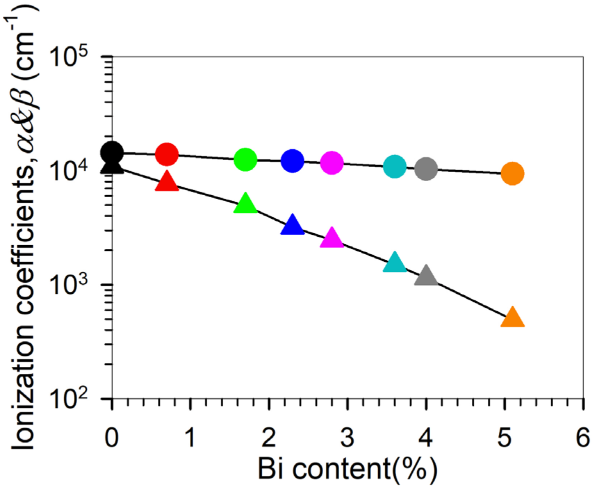

Fig. S11 α (circles) and β (triangles) as functions of Bi content at an inverse electric field of 3×10^{-6} cm/V. The black solid lines are guides to the eye.

References

[1] Richards, R. D. *et al.* Molecular beam epitaxy growth of GaAsBi using As_2 and As_4. *J. Cryst. Growth* **390***,* 120-124 (2014)

[2] Janotti, A., Wei, S. H. & Zhang, S. B. Theoretical study of the effects of isovalent coalloying of Bi and N in GaAs. *Phys. Rev. B* **65**, 115203 (2002)

[3] Dresselhaus, M. S. Solid State physics part ii optical properties of solids. *Lecture Notes (Massachusetts Institute of Technology, Cambridge, MA)* **17**, (2001)

[4] Broderick, C. A., Usman, M. & O'Reilly, E. P. Derivation of 12- and 14-band k·p Hamiltonians for dilute bismide and bismide-nitride semiconductors. *Semicond. Sci. Technol.* **28,** 125025 (2013).

[5] Usman, M. *et al.* Tight-binding analysis of the electronic structure of dilute bismide alloys of GaP and GaAs. *Phys. Rev. B* **84**, 245202 (2011)

[6] Sweeney, S. J. & Jin, S. R. Bismide-nitride alloys: Promising for efficient light emitting devices in the near- and mid-infrared. *J. Appl. Phys.* **113**, 043110 (2013)

[7] Gelczuk, Ł. *et al.* Deep-level defects in n-type GaAsBi alloys grown by molecular beam epitaxy at low temperature and their influence on optical properties. *Sci. Rep.* **7**, 12824 (2017)

[8] Mohmad, A. R. *et al.* Localization effect and band gap of GaAsBi alloys. *Phys. Status Solidi B* **251**, 1276-1281 (2014)

[9] Huang, W. & Oe, K. Molecular-beam epitaxy and characteristics of $GaN_yAs_{1-x-y}Bi_x$. *J. Appl. Phys.* **98**, 053505 (2005)

[10] Tixier, S. *et al.* Molecular beam epitaxy growth of $GaAs_{1-x}Bi_x$. *Appl. Phys. Lett.* **82**, 2245 (2003)

[11] Francoeur, S. *et al.* Band gap of $GaAs_{1-x}Bi_x$, 0<x<3.6%. *Appl. Phys. Lett.* **82**, 3874 (2003)

[12] Yoshida, J., Kita, T., Wada, O. & Oe, K. Temperature Dependence of $GaAs_{1-x}Bi_x$ Band Gap Studied by Photoreflectance Spectroscopy. *Jpn. J. Appl. Phys.* **42**, 371 (2003)

[13] Alberi, K. *et al.* Valence band anticrossing in $GaAs_{1-x}Bi_x$. *Appl. Phys. Lett.* **91**, 051909 (2007)

[14] Batool, Z. *et al.* The electronic band structure of GaAsBi/GaAs layers: Influence of strain and band anti-crossing. *J. Appl. Phys.* **111**, 113108 (2012)

[15] Lau, K. S. *et al.* Excess noise measurement in avalanche photodiodes using a transimpedance amplifier front-end. *Meas. Sci. Technol.* **17**, 1941 (2006)

[16] Woods, M. H., Johnson, W. C. & Lampert, M. A. Use of a Schottky barrier to measure impact ionization coefficients in semiconductor. *Solid-State Electron.* **16**, 381-394 (1973).

[17] David, J. P. R., Marsland, J. S. & Roberts, J. S. The electron impact ionization rate and breakdown voltage in GaAs/$Ga_{0.7}Al_{0.3}As$ MQW structures. *IEEE Electron Device Lett.* **10**, 294-296 (1989)
